# An idiographic network approach to modeling daily loneliness and paranoia in psychosis: implications for personalized interventions

**DOI:** 10.3389/fpsyt.2026.1759800

**Published:** 2026-05-13

**Authors:** Jakub Januška, Daniel Dančík, Michal Hajdúk

**Affiliations:** 1 Department of Psychology, Faculty of Arts, Comenius University in Bratislava, Bratislava, Slovakia; 2 Psychiatric Clinic, Faculty of Medicine, Slovak Medical University, Bratislava, Slovakia; 3 Department of Psychiatry, Faculty of Medicine, Comenius University in Bratislava, Bratislava, Slovakia

**Keywords:** experience sampling method, idiographic approach, loneliness, network analysis, paranoia

## Abstract

The call for personalized approaches in psychopathology is growing, as group-level analyses fail to capture the person-specific patterns. This study used an idiographic network approach to model the daily dynamics of loneliness, paranoia, affect, and social motivation in seven individuals with psychosis. We applied Group Iterative Multiple Model Estimation (GIMME) to intensive longitudinal data collected using the Experience sampling method. Results revealed substantial inter-individual heterogeneity, particularly in social motivation pathways and the role of paranoia in affective changes. Specifically, the relationship between social avoidance and approach was positive for one participant and negative for another. Furthermore, a unique temporal effect of paranoia predicting subsequent negative affect was identified in one case. These findings highlight the critical relevance of idiographic methods for moving beyond generalized models and identifying precise, actionable targets for developing tailored interventions in psychosis.

## Introduction

Loneliness, a perceived discrepancy between one’s desired and actual level of social connection (1), is a critical public health concern linked to numerous adverse mental and physical health outcomes (2–4). It is particularly pronounced in psychotic disorders, where it contributes to reduced social functioning and poorer health outcomes (5–7). Paranoia, a central symptom of psychosis, shows a consistent association with loneliness (8–10). An evolutionary perspective suggests that lonely individuals also feel unsafe and experience heightened sensitivity to threat, which is a core feature of paranoia (11). Another hypothesis suggests that social contact has a correcting or distracting effect on paranoid beliefs – a benefit less available to individuals with reduced social connections (12). Conversely, paranoid thinking inherently fosters mistrust, which can lead to safety behaviors like social avoidance or withdrawal, suggesting there is a vicious cycle between loneliness and paranoia (1, 13, 14). Several studies have investigated the temporal association between loneliness and paranoia, however, this bidirectional relationship is not observed consistently (15–18), and these patterns can vary greatly across individuals. Therefore, taking idiographic perspective into account is essential.

Another feature shared by both loneliness and paranoia is their clear association with negative affect (8, 19, 20). Empirically supported cognitive models of persecutory delusions posit a vicious cycle between paranoia and negative affect, wherein emotional distress plays a critical role in both the emergence and maintenance of paranoid beliefs (13, 21). Looking at loneliness, it is most strongly associated with depression (2). In addition, loneliness appears uniquely related to social motivation (1, 22). Specifically, transient experiences of increased loneliness are expected to boost motivation to engage in social interaction (social approach) and lower the desire to withdraw from social situations (social avoidance), in an adaptive response to re-establish social connection. However, this mechanism can turn maladaptive when loneliness becomes chronic (23, 24). This means that the role of social motivation in this context can be different across individuals.

While most studies in the field have used cross-sectional designs and focused on between-person effects, there is a growing need for a personalized approach in psychopathology research. Because psychological processes like paranoia and loneliness are highly dynamic and heterogeneous, they violate the assumption of ergodicity (25). This means that the structure of associations found at the between-person level cannot be automatically applied to the within-person processes of a specific individual. To truly understand how these symptoms unfold in an individual over time, employing idiographic methods is essential.

A growing body of research uses intensive longitudinal approaches, such as the Experience sampling method (ESM), to examine temporal dynamics of social connection over days or weeks (1, 19). This approach supports idiographic conceptualization that prioritizes the unique processes within individuals, thereby enabling the development of personalized models of psychopathology (26, 27). Furthermore, a network approach has been successfully applied to loneliness and paranoia research, including studies using ESM data (14, 28, 29). Among the available modeling techniques for idiographic ESM data, Group Iterative Multiple Model Estimation (GIMME; 30) represents a powerful person-specific tool that simultaneously accounts for patterns present across the entire sample and includes lagged relationships. To the best of our knowledge, no studies up to this date have attempted to estimate individual dynamics linking paranoia and loneliness with positive and negative affect, and aspects of social motivation. Therefore, this study applies GIMME to model the unique associations within these variables with the primary focus on the person-specific dynamics over time. This approach of data visualization through person-specific networks holds significant clinical potential, as it can help shared decision-making and tailor personalized interventions based on established cognitive models of psychosis.

## Methods

The current analysis included seven clinician-referred outpatients (five women) with a confirmed diagnosis of schizophrenia spectrum disorders. Participant ages ranged from 23 to 47 years. They were enrolled as a part of a larger study examining social processes in autism and schizophrenia, which involved a laboratory session featuring a comprehensive battery of behavioral, physiological, and psychological assessments with an optional ESM follow-up. The detailed clinical and demographic description of each participant is displayed in **Table 1**. The participants are hereafter referred in text as P1 through P7.

**Table 1:** Detailed clinical and demographic description of individual participants.

Participant	Sex	Symptom severity		Education	Employment
		BPRS Total	BPRS Susp.		
P1	F	35	1	Master’s degree	Disability pension + employed
P2	F	51	3	High school	Part-time job
P3	F	46	4	High school	Student + part-time job
P4	F	37	1	Master’s degree	Part-time job
P5	M	33	1	High school	Student + part-time job
P6	M	39	2	Master’s degree	Student + part-time job
P7	F	66	4	Bachelor’s degree	Student + part-time job

Symptom severity was assessed using the 24-item Brief Psychiatric Rating Scale (BPRS), with total scores ranging from 24 to 168. BPRS Susp., BPRS Suspiciousness score; F, female; M, male.

Upon consenting to the ESM phase, participants completed a questionnaire comprising 35 multiple-choice items. They received eight notifications daily for six consecutive days, for a total of 48 notifications, delivered according to a semi-randomized schedule within predefined time intervals across the day. The maximum theoretical time interval between two notifications within a day was 4 hours. The minimum time interval was not restricted. Loneliness, social avoidance, and social approach were each measured by a single item (“I feel lonely,” “I avoid other people,” “I am interested in being with other people”). Paranoia was calculated as a composite score of three ESM items (“I feel that others might hurt me,” “I feel suspicious,” “I feel that I need to be on guard against others.”). Negative affect and positive affect were each measured using four-item composites (negative affect: “I feel sad,” “ashamed,” “irritated,” “anxious”; positive affect: “I feel good,” “excited,” “relaxed,” “cheerful”), each reflecting momentary emotional states. Composite scores were calculated as simple means. If a participant missed any item at a specific time point, the corresponding composite score was treated as missing. Participant compliance was high, with completion rates ranging from 79% to 94% of all notifications.

Data were analyzed in R version 4.5.1 using *gimme* package (30) to model a separate network for each participant. Each network displays both contemporaneous and temporal (t-1 lagged) effects. GIMME was selected over more traditional methods, such as multilevel Vector Autoregression (mlVAR), because it does not force individuals to conform to a group-level structure if it does not fit their data (avoiding shrinkage), thereby prioritizing true idiographic discovery. Additionally, unlike mlVAR, GIMME utilizes a unified Structural Equation Modeling (uSEM) framework, which allows for the estimation of directed contemporaneous effects. The individual network models included six variables and were constrained to a fixed hexagonal layout for easier visual comparison across the networks. To retain all potentially meaningful pathways, the group-level paths cutoff parameter was set to identify connections common to at least three of the seven participants, and the alpha level for the individual-level path pruning step was set at 0.05. Missing observations were handled natively during model estimation by the GIMME algorithm, without conducting a prior manual data imputation strategy.

## Results

**Figure 1:**
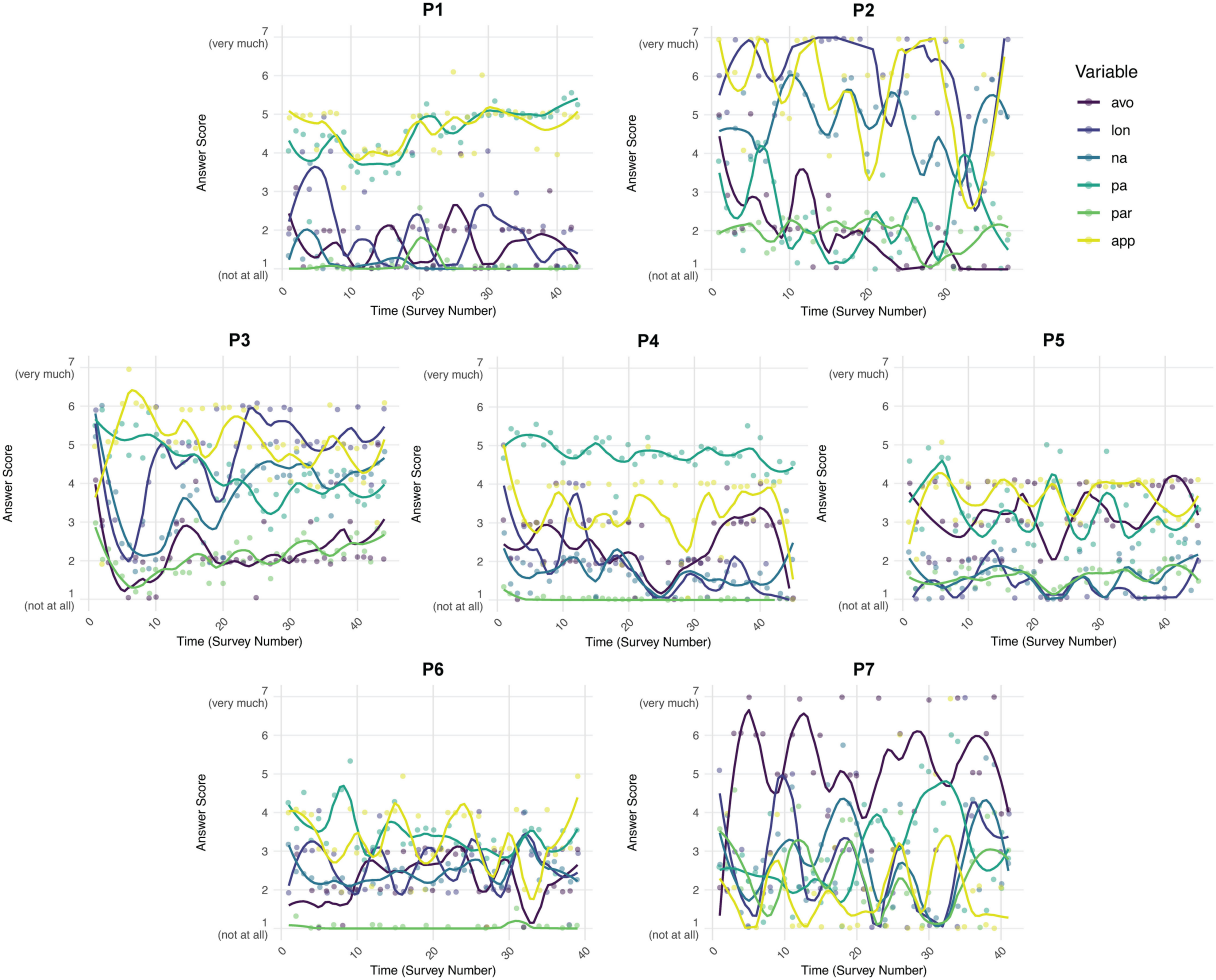
Descriptive plots of the studied variables development over time for the seven participants (P1-P7). P1 – female, 39, P2 – female, 28, P3 – female, 25, P4 – female, 47, P5 – male, 39, P6 – male, 45, P7 – male, 23. avo, social avoidance; lon, loneliness; na, negative affect; pa, positive affect; par, paranoia; app, social approach.

The temporal dynamics of the six observed variables for each participant are depicted in **Figure 1**. Rich inter-individual variability in these variables was observed across the sample. The patterns of loneliness and paranoia levels and fluctuation varied substantially between individuals.

**Figure 2:**
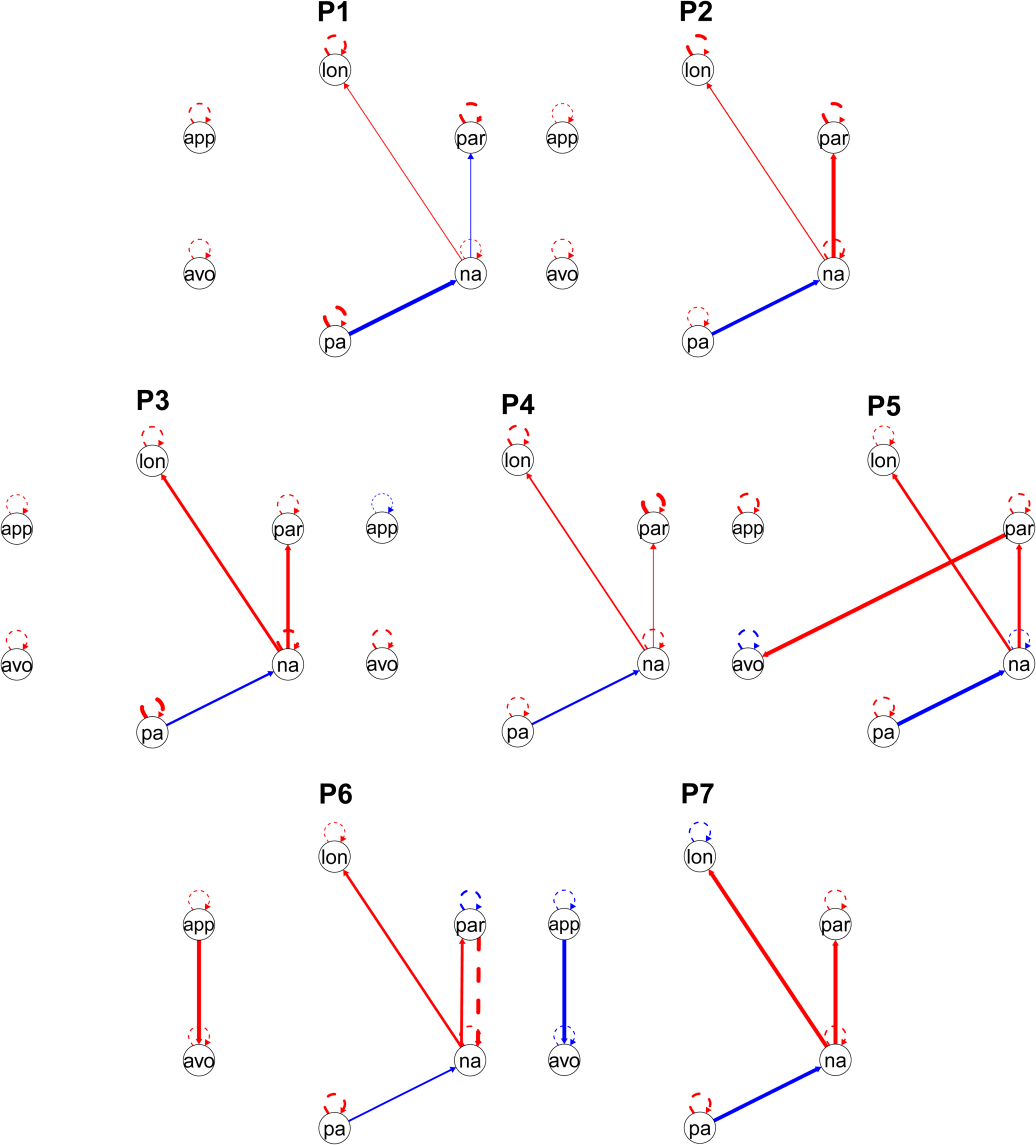
Idiographic networks for seven participants (P1-P7). Red lines indicate positive associations, and blue lines indicate negative associations. Solid lines indicate directed contemporaneous effects, and dashed lines indicate temporal (t-1 lagged) effects. avo, social avoidance; lon, loneliness; na, negative affect; pa, positive affect; par, paranoia; app, social approach.

The network models are displayed in **Figure 2**. Each network displays combined directed contemporaneous effects (within the same time point), and temporal effects (lagged t-1 state predicting a subsequent assessment). Several common pathways emerged across the sample, alongside a few unique person-specific connections. The initial group-level search identified 12 paths (including the autoregressive effects) which met the threshold for inclusion in the person-specific network estimation. Negative affect consistently demonstrated a positive association with loneliness and paranoia and a negative association with positive affect. Specifically, paranoia was positively linked to social avoidance in one participant. Social avoidance was further associated with social approach in two participants, albeit with opposing directionality (i.e., one positive and one negative connection).

The identified associations were predominantly contemporaneous. A single temporal relationship was detected, with paranoia preceding subsequent higher negative affect in one participant. Finally, all models included significant autoregressive effects, though they were not uniformly positive. Negative autoregressive effects were observed in one participant for loneliness, negative affect, and paranoia, and in two participants for social avoidance, and social withdrawal.

## Discussion

The analysis revealed several person-specific patterns, as well as common dynamics present in most of the participants. The unique patterns have implications on the specific approach that might be needed for specific patients.

The most prevalent associations across the sample involved negative affect, which functioned as a key bridging element between loneliness, paranoia, and positive affect. The role of negative affect in linking loneliness and paranoia has been previously described in general population (8, 31). Similar patterns have been found also in patients with a psychotic disorder (19, 32). Given its key role in all networks, it is possible that negative affect acts as a momentary mediator between loneliness and paranoia. Loneliness may not directly precede threat beliefs, but instead increase negative affect, which can in turn trigger paranoid thoughts. Furthermore, in P6, this pattern was strengthened by a lagged effect of paranoia on negative affect. Paranoid thoughts not only co-occurred with emotional distress, but the effect of paranoia on emotional distress persisted even several hours later. This dynamic suggests that for P6, paranoia demonstrates a strong spillover effect on the affective system, potentially indicating a pattern of reduced emotional flexibility and a persistent, maladaptive impact of paranoid thoughts on mood. Clinically, this makes timely intervention on paranoid thoughts crucial. On the other hand, one of our participants differed from the rest of the sample in the polarity of the association between paranoia and negative affect. In P1, the association was negative, meaning that increased paranoia was accompanied by a decrease in negative affect at the same time point. This unusual pattern suggests a lack of emotional distress typically accompanying persecutory thoughts (32). Importantly, an examination of the descriptive data reveals that P1 experienced relatively low overall levels of paranoia throughout the sampling period. Therefore, this negative association may as well reflect a floor effect rather than an emotional disconnection, indicating that the paranoid thoughts were mild and did not trigger severe distress. Regardless of the cause, this pattern stands in contrast to P6, where paranoia functioned as a predictor of future distress. Viewing these results through the lens of the cognitive model of paranoia (13, 21), this heterogeneity suggests that for some patients, emotional distress is a primary maintaining factor for paranoia requiring immediate targeted intervention. For others experiencing mild or less distressing paranoia, the clinical focus may need to shift away from emotion regulation and toward cognitive reappraisal of the threat itself. A possible clinical intervention for P1 might explore the nature of paranoid thoughts and patient’s coping strategies to create personalized therapeutic interventions that focus more on cognitive strategies rather than emphasizing the emotional component of paranoia.

Our results further show that social motivational dynamics are highly person-specific and represent a strong candidate for tailored personalized interventions. In P5, social avoidance was positively associated with paranoia, suggesting a pattern of response to a threat, where paranoid thoughts trigger a conscious urge for social withdrawal. However, in this case it does not seem like a typical, stable state of withdrawal. P5 showed a unique negative autoregressive path on social avoidance, which might potentially reflect an active self-regulatory process that disrupts the continuity of social avoidance in time. In that case, a suggested effective strategy for P5 could be to directly target paranoid thoughts, weakening the path toward social avoidance, while simultaneously reinforcing the patient’s inherent self-correcting tendencies. At the same time, it is plausible that the negative autoregressive effect reflects high system instability or a rapid fluctuation not captured by the selected measurement window, requiring more investigation before choosing a suitable intervention. Going further, the observed relationships between social avoidance and social approach provide an insight into person-specific motivational dynamics. For P6, this association was positive, meaning that avoidance was simultaneously accompanied by a drive to approach. This pattern suggests a state of conflicted social ambivalence rather than simple disinterest. Consequently, a potential intervention for P6 might primarily target social avoidance causing a disruption in social engagement to support the co-existing motivation to connect. In contrast, the negative connection between social avoidance and social approach in P7 indicates a more decisive, unambiguous motivational state. Crucially, the negative autoregressive effects for both variables reveal a system with low inertia, where states of high avoidance and low approach are inherently transient. This fluctuation might function as a self-preservation strategy when social contact might become overwhelming. A therapeutic intervention for P7 might attempt to understand this cycle and help maximize the quality of the high-motivation phases while respecting the potentially restorative function of the phases with low motivation. Similarly to the above, an alternative interpretation of the negative autoregressive path is an unstable and dysregulated motivational system. In that case, an effective intervention would require more in-depth investigation.

We did not observe significant connections between loneliness and social approach and avoidance. This appears inconsistent with the social homeostasis theory, which posits that loneliness acts as a regulatory signal to activate social motivation and restore a desired level of social connection (1, 24). Notably, the social homeostasis theory was derived from, and primarily studied in, healthy populations. However, it has been proposed that this adaptive, compensatory response can become inherently disrupted in cases of chronic loneliness (23), a condition that occurs more frequently in individuals with psychosis. Consequently, stable, trait-level deficits in social motivation, rather than dynamic, state-dependent fluctuations, might offer a more direct explanation for the absence of this regulatory behavior in this population. Future research could investigate this by combining ESM designs with baseline trait measures of chronic loneliness to disentangle trait-like deficits from momentary processes.

Several limitations should be considered when interpreting the current results. The small sample size, combined with high data variability, may have contributed to the fact that some commonly observed between-person associations, especially the direct relationship between loneliness and paranoia, have not been captured, as well as the small proportion of temporal relationships observed. Understandably, the current sample does not allow for generalization of the observed relationships to a broader population. Another limitation is the use of single-item measures for loneliness, social approach, and social avoidance, which might have constrained the operationalization of these constructs. Furthermore, the current idiographic models capture a narrow snapshot of a 6-day sampling window, which might not be sufficient to expect these network structures to be stable over a longer time period. Therefore, the offered suggestion for therapeutic strategies should be understood with caution. Finally, we did not assess for chronic loneliness, which could have strengthened our interpretation of the absent link between state loneliness and social motivation.

In conclusion, these findings illustrate the substantial inter-individual similarity and variability that can be expected even among individuals with schizophrenia spectrum disorders. We show that while negative affect remains a critical mechanism for understanding the processes associated with loneliness and related psychopathology in schizophrenia, the specific link between negative affect and paranoia can vary substantially in intensity. Furthermore, social motivation pathways, particularly the relationships between social avoidance, paranoia, and social approach, exhibited considerable person-specific heterogeneity. We stress the importance of employing the idiographic approach to support personalized interventions.

## Data Availability

The raw data supporting the conclusions of this article will be made available by the authors, without undue reservation.
